# Proteomics Analysis of Aqueous Humor and Rejected Graft in Pig-to-Non-Human Primate Corneal Xenotransplantation

**DOI:** 10.3389/fimmu.2022.859929

**Published:** 2022-03-24

**Authors:** Jae Won Oh, Chang Ho Yoon, Jin Suk Ryu, Kwang Pyo Kim, Mee Kum Kim

**Affiliations:** ^1^ Department of Applied Chemistry, Institute of Natural Science, Global Center for Pharmaceutical Ingredient Materials, Kyung Hee University, Yongin, South Korea; ^2^ Department of Biomedical Science and Technology, Kyung Hee Medical Science Research Institute, Kyung Hee University, Seoul, South Korea; ^3^ Laboratory of Ocular Regenerative Medicine and Immunology, Seoul Artificial Eye Center, Seoul National University Hospital Biomedical Research Institute, Seoul, South Korea; ^4^ Department of Ophthalmology, Seoul National University College of Medicine, Seoul, South Korea; ^5^ Department of Ophthalmology, Seoul National University Hospital, Seoul, South Korea; ^6^ Transplantation Research Institute, Seoul National University Medical Research Center, Seoul, South Korea

**Keywords:** proteomics, cornea, xenotransplantation, aqueous humor, rejection, pig, non-human primate (macaque)

## Abstract

Although pig-to-non-human primate (NHP) corneal xenotransplantation has shown long-term graft survival, xenogeneic antigen-related immune responses are still stronger than allogeneic antigen-associated responses. Therefore, there is an unmet need to investigate major rejection pathways in corneal xenotransplantation, even with immunosuppression. This study aimed to identify biomarkers in aqueous humor for predicting rejection and to investigate rejection-related pathways in grafts from NHPs transplanted with porcine corneas following the administration of steroids combined with tacrolimus/rituximab. NHPs who had received corneas from wild-type (WT) or α-1,3-galactosyltransferase gene-knockout (GTKO) pigs were divided into groups with or without rejection according to clinical examinations. Liquid chromatography-mass spectrometry (LC-MS) was used to analyze the proteomes of corneal tissues or aqueous humor. The biological functions of differentially expressed proteins (DEPs) were assessed using Gene Ontology (GO) and Kyoto Encyclopedia of Genes and Genomes (KEGG) for pathways and protein–protein interaction network analysis. Among the 66 DEPs in aqueous humor, complement proteins (C3, C5, and C9) and cholesterol metabolic proteins (APOA1 and APOA2) were related to xenogeneic rejection as biomarkers, and alternative pathways of the complement system seemed to be important in xenogeneic graft rejection. Among the 416 DEPs of the cornea, NF-κB1 and proteosomes (PSMD7, PSMA5, and PSMD3) seemed to be related to xenogeneic graft rejection. Additionally, oxidative phosphorylation and leukocyte activation-related pathways are involved in rejection. Overall, our proteomic approach highlights the important role of NF-κB1, proteosomes, oxidative phosphorylation, and leukocyte activation-related inflammation in the cornea and the relevance of complement pathways of the aqueous humor as a predictive biomarker of xenogeneic rejection.

## 1 Introduction

Corneal allotransplantation is the standard surgical treatment for medication-resistant corneal blindness (1). Although corneal allotransplantation is widely applied in certain areas, there is still an unmet need for corneal blindness treatments given the donor shortage in developing countries (2). Corneal xenografts or bioengineered products can be considered as alternatives or substitutes for corneal allografts (3). However, xenogeneic antigen-related immune responses are stronger than allogeneic antigen-associated immune responses (3). To overcome hyperacute rejection, which is mediated by natural anti-Galα1-3Galβ1-4GlcNAc-R (anti-αGal) antibodies, alpha-1,3-galactosyltransferase gene-knockout (GTKO) pigs are used in xenotransplantation (4). Nevertheless, corneal pig-to-non-human primate xenotransplantation using GTKO donors still requires extensive immunosuppression (5). Therefore, there are still unmet needs for corneal xenotransplantation given their unknown xenogeneic antigen’s strong immune response.

Recently, LC-MS-based proteomics has evolved to detect low abundant critical proteins in various diseases and allows possible longitudinal monitoring in small-volume samples (6). LC-MS-based proteomics is useful in corneal transplantation research in which the amount of biological specimens is limited, such as the cornea and aqueous humor (AH). To identify biological markers during the rejection of islet allografts placed in the anterior chamber, a small amount of AH can be used for longitudinal proteomics analysis (7). In addition, to identify non-αGal antigens in pig-to-primate cardiac xenotransplantation, a proteomic approach has been applied (8).

Proteomic approaches have shown that neutrophil-mediated immune processes and complement-related and endoplasmic reticulum–phagosome pathways may be critical inflammatory surveillance responses in normal human corneas (9). High concentrations of aqueous interleukin (IL)-6, IL-17A, interferon (IFN)-γ, monocyte chemotactic protein (MCP)-1, soluble intercellular adhesion molecule (sICAM)-1, and interferon gamma-induced protein 10 (IP-10) have been identified as biomarkers for predicting corneal allograft rejection (10, 11). Tear coronin-1A has also been reported as a biomarker of acute corneal rejection in rats (12). However, rejection-predicting biomarkers remain inconclusive in corneal allotransplantation (13). Given that CD8^+^IFNγ^+^ T cells and the aqueous complement protein C3a were discovered to predict rejection in corneal xenotransplantation (14), a whole proteomic approach is required to analyze interactive immune pathways and their evolution with the progression of rejection. Therefore, this study aimed 1) to investigate proteomic changes in rejected xenocorneal grafts even with immunosuppressive agents, 2) to identify aqueous biomarkers with xenogeneic rejection progression in pig-to-NHP corneal xenotransplantation, and 3) to evaluate whether xenogeneic rejection-networking pathways would be different between wild-type (WT) and GTKO corneal grafted NHPs.

## 2 Materials and Methods

This study adhered to the ARVO Statement regarding the Use of Animals in Ophthalmic and Vision Research. This study was approved by Seoul National University (SNU) (IACUC: SNU‐151102‐3) and SNU Hospital (IACUC: 15‐0171, 18-0160). Human corneas (*n* = 4) rejected after corneal transplantation due to bullous keratopathy were obtained during re-corneal transplantation.

### 2.1 Study Design

**Figure 1:**
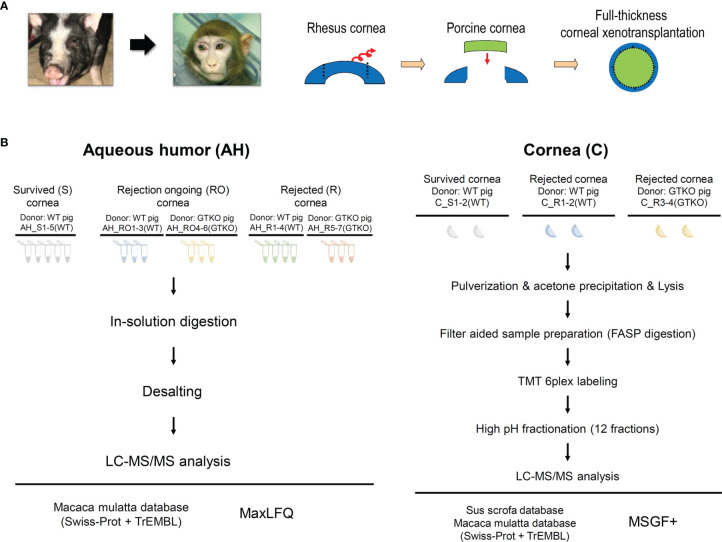
**(A)** Schematic diagram depicting the procedure used for full-thickness corneal xenotransplantation. A clinically applicable porcine corneal donor button of 7.5 mm was used on the 7.0-mm non-human primate recipient bed in all cases. **(B)** Overall experimental workflow of the proteomics experiments. Aqueous humor (AH) and cornea were lysed and digested into peptides. Digested peptides were analyzed using LC-MS/MS. AH samples were subjected to LFQ analysis and cornea samples were subjected to TMT quantitative analysis. WT, wild type; GTKO, α-1,3-galactosyltransferase gene-knockout; LC, liquid chromatography; LFQ, label-free quantification; TMT, tandem mass tag.

We investigated a cohort of 25 rhesus macaques that had undergone full-thickness WT (*n* = 20) or GTKO (*n* = 5) miniature pig corneal xenotransplantation between 2016 and 2018 (5, 14–16). Penetrating keratoplasty procedures were described in previous studies (**Figure 1A**) (5, 15). After transplantation, a physical examination was performed weekly to calculate the graft score, and the AH was collected every 2–4 weeks. The corneal graft score (0–12) was calculated based on opacity, edema, and vascularization, as described previously (16). Rejection was defined as a score of ≥6. Rejection ongoing (RO) was defined as a score of 4 or 5 with increasing scores over the past 2 weeks. Non-rejected survival control was defined as a score of 0 at least 6 months (180 days) after transplantation. Recipients diagnosed with graft rejection were sacrificed within 2 weeks. Recipients with survived grafts were monitored for up to 6 months. This proteomics analysis was performed on 18 AH and 6 corneal samples from the cohort recipients. Samples were stored at −80°C until analysis. Donor pig characteristics, sample acquisition times, and graft scores of the groups (survival, RO, and rejection) are summarized in **Table 1**. Recipients were administered systemic and topical immunosuppressants, as listed in **Supplementary Table 1**.

**Table 1:** Summary of the groups used for corneal and aqueous proteomic analyses.

Group name	Number	Donor	Sample acquisition time (after transplantation, weeks)	Graft score
Aqueous humor analysis				
Survival (S)	5	WT	19, 20, 20, 21, 22	0
Rejection ongoing (RO)	3	WT	8, 13, 21	4 or 5
	3	GTKO	5, 10, 21	4 or 5
Rejection (R)	4	WT	5, 18, 23, 24	6
	3	GTKO	8, 12, 18	6
Corneal analysis				
Survival	2	WT	26, 67	0
Rejection	2	WT	22, 25	6
	2	GTKO	19, 23	6

The AH samples at week 5 from the GTKO rejection ongoing group and week 8 from the GTKO rejection group and the AH samples at week 10 from the GTKO RO group and at week 12 from the GTKO rejection group were from the same recipients. All the other samples were obtained from different recipients.

WT, wild type; GTKO, α-1,3-galactosyltransferase gene knockout.

### 2.2 AH Sample for Analysis

Approximately 140 µl of AH was collected and divided in half. Half of the samples were used for proteomics analysis and the other half were used for biomarker candidate assays. The 18 analyzed AH samples were stratified as follows: 1) survival (control) group (*n* = 5), AH at the nearest 20 weeks after transplantation, all recipients were transplanted with WT porcine cornea; 2) RO group (*n* = 6), AH at graft scores of 4 or 5, three recipients were transplanted with WT porcine cornea and the other three with the GTKO porcine cornea; and 3) rejection group (*n* = 7), AH at graft scores of 6, four recipients were transplanted with WT porcine cornea and the other three with the GTKO porcine cornea (**Figure 1B**).

### 2.3 Corneal Xenotransplantation Tissue for Analysis

After sacrifice, the entire cornea of the recipient NHP was excised, including the limbus. Thus, the central circular part of the tissue was the porcine cornea, and the tissue surrounding this region was the cornea of the NHP recipient. Half of the excised tissues (semicircular shape) were used for proteomics analysis. The six analyzed corneal samples were stratified as follows: 1) survival (control) group (*n* = 2), in which all recipients were transplanted with WT porcine cornea, and 2) rejection group (*n* = 4), in which two recipients were transplanted with WT porcine cornea and the other two with the GTKO porcine cornea (**Figure 1B**).

### 2.4 Proteomics Analysis

#### 2.4.1 Aqueous Humor

To study AH proteins characterized by low protein concentrations, we adopted direct in-solution digestion and label-free quantification (LFQ) analysis to minimize protein loss during the experiments. Each AH sample was collected and the protein concentration of each sample was measured using the BCA assay (Pierce, Rockford, IL, USA). An equal amount of protein of each AH sample (20 μg) was digested into peptides using 8 M urea in 100 mM ammonium bicarbonate (Sigma, St. Louis, MO, USA) incubated for 20 min at room temperature (RT). Reduction using 10 mM dithiothreitol (Sigma) and alkylation with 30 mM iodoacetamide (Sigma) were held to denature the proteins. Trypsin was used to treat the sample [with a 1:50 (trypsin:sample) ratio] and incubated at 37°C for 12 h. To quench the trypsin activation, 0.4% TFA was added to the sample and the digested peptides were desalted using the C18 spin column (Pierce). The desalted peptides were dried and resuspended with sol A (0.1% formic acid) for the next step—LC-tandem mass spectrometry (MS/MS).

A high-throughput and high-resolution mass spectrometer, Q Exactive Orbitrap Hybrid Mass Spectrometer coupled with the EASY-nLC 1000 system (Thermo Scientific, Bremen, Germany), was adopted to identify as many proteins as possible. Three-hour gradients (5%–40% of sol B for 130 min, 40%–80% of sol B for 5 min, holding at 80% of sol B for 10 min and equilibrating at 1% of sol B for 30 min; sol A: 0.1% FA in water, sol B: 0.1% FA in acetonitrile) were used. Full MS with scan range (400–2,000 *m*/*z*) was acquired in a resolution of 70,000 at *m*/*z* 200. Automated gain control (AGC) target value was targeted to 1.0 × 10^6^ with a maximum ion injection time of 120 ms. The maximal ion injection time for MS/MS was set to 60 ms with a resolution of 17,500. To acquire various MS/MS features, dynamic exclusion time was set to 30 s.

A software platform for LC-MS/MS shotgun proteomics, MaxQuant, was used to identify the AH proteins with the UniProt *Macaca mulatta* database (2021.09 released. Reviewed + TrEMBL) (17). At least two unique and razor peptides were selected to represent a protein. A fixed modification of carbamidomethylation on cysteine and oxidation on methionine as dynamic modification was counted on the LFQ search. In the peptide spectrum match and protein levels, a 1% false discovery rate (FDR) cutoff was applied. Mass tolerance for precursor was up to 4.5 ppm and fragment mass tolerance was up to 20 ppm. The “match between runs” option was used for retention time alignment and identifying potential mismatched proteins; 0.7 min match time window and 20 min alignment time window were set for the identification.

LFQ intensity was processed with R software (v. 4.0.2). Potential contaminants were removed (reversed database, contaminants, only identified by site) and the intensity of the protein left was converted into log2 value and statistically analyzed. Differentially expressed proteins with strict cutoff (2-fold change, 0.05 < *p*-value from independent *t*-test) were enriched and used for further analysis.

#### 2.4.2 Corneal Tissue

The cornea is composed of keratocytes and extracellular matrix (ECM) with collagens. Therefore, unlike AH, the cornea was individually cryopulverized using a cryoPREP device (CP02, Covaris, Woburn, MA, USA) and dissociated in lysis buffer (4% SDS, 0.1 M Tris–HCl, pH 7.6) with sonication using a focused ultrasonicator. Twenty sonication cycles were repeated at 16°C. The homogenate was centrifuged at 16,000*g* at 20°C for 10 min. The supernatant was collected and the protein concentration was measured using the same procedure as the AH sample. The lysed sample was digested using the filter-aided sample preparation (FASP) method (18). An equal amount of the sample (100 μg) with SDT buffer (4% SDS in 0.1 M Tris–HCl, pH 7.6, and 0.1 M DTT) was transferred to a Microcon filter tube (YM-30, Millipore Corporation), and 8 M urea in 0.1 M Tris–HCl, pH 8.5, was mixed with the sample and centrifuged at 14,000*g* for 60 min to remove SDS. After alkylation with 0.05 M iodoacetamide in 8 M urea for 30 min, a 1:50 trypsin:sample ratio was treated at 37°C overnight. The digested peptides were dried and resuspended with 10 mM triethylammonium bicarbonate (TEAB) in water (pH 7.5) for labeling tandem mass tag (TMT) reagents (19). A six-plex TMT was labeled following the instructor’s guide [C_R4(GTKO)—126, C_R3(GTKO)—127, C_R2(WT)—128, C_R1(WT)—129, C_S1(WT)—130, C_S2(WT)—131]. The labeled peptides were pooled together and dried for the next step—high pH fractionation. The dried peptides were suspended and underwent high pH fractionation. The XBridge C18 analytical column (4.6 mm × 250 mm, 130 Å, 5 mm) was used for the peptide separation. Sol A (10 mM ammonium formate, pH = 10) and sol B (10 mM ammonium formate in 90% ACN, pH = 10) were used as the mobile phase. The gradient was as follows: 0~10 min, 5% B; 10~70 min, 35% B; 70~80 min, 70% B; 80~85 min, 70% B; 85~90 min, 5% B; and 90~105 min, 5% B. Peptides were separated into 12 fractions and the separated peptides were dried in a SpeedVac and desalted with a C18 spin column.

Since the cornea peptides were labeled with TMT reagents, different MS settings were used. To acquire the reporter ion *m*/*z*, the fixed first *m*/*z* was set to 100 *m*/*z* on MS/MS scan. The rest of the values are the same as the AH LC-MS/MS analysis.

Raw files from LC-MS/MS analysis proceeded with postexperiment monoisotopic mass refinement (PEMMR) to increase sensitivity in peptide identification and accuracy by selecting the unique mass class (UMC). MSGF+ was used to find proteins using two species (*Macaca mulatta*, *Sus sucrofa*) from the UniProt database (2021.09 released. Reviewed + TrEMBL). In modification, carbamidomethylation on cysteine and TMT six-plex modification of lysine, N-termination as static modification, and oxidation of methionine as a variable modification were used. The 1% FDR for peptide level was adopted. Unlike MaxQuant LFQ analysis, TMT quantitative analysis used the reporter ion intensity to quantify the proteins. The exported reporter ions were normalized and used for statistical analysis. Since TMT quantitative analysis reduced the variation during the experiments, different cutoff values were applied to the cornea proteomics. Differentially expressed proteins with cutoff (1.5-fold change, 0.05 < *p*-value from independent *t*-test) were enriched and used for further analysis.

### 2.5 Biomarker Candidates of AH Assay by ELISA

C3a, a complement activation product, was chosen as a biomarker candidate of AH related to corneal xenograft rejection. The levels of C3a in the AH were measured using the OptEIA™ Human C3a ELISA Kit (BD Biosciences) according to the manufacturer’s protocol. The upper detection limit of C3a concentration was 25 ng/ml.

### 2.6 Statistical and Bioinformatics Analyses

Gene Ontology (GO) search and Kyoto Encyclopedia of Genes and Genomes (KEGG) pathway mapping were performed using g:Profiler (20). Less than 0.05 *p*-value GO terms were enriched and used for constructing a network. The STRING public database was used for the protein-to-protein interaction network model, and the network was built with sorted DEPs and interactome data using Cytoscape (21). For multiple comparisons, Kruskal–Wallis test followed by Dunn’s *post-hoc* test was used with statistical significance at *p <*0.05. All the statistical calculations and plots were made using R (v. 4.0.2) or GraphPad Software (version 9.3.1; GraphPad Software, La Jolla, CA, USA).

## 3 Results

### 3.1 Proteomics Analysis of the NHP Aqueous Humor During Xenograft Rejection

**Figure 2:**
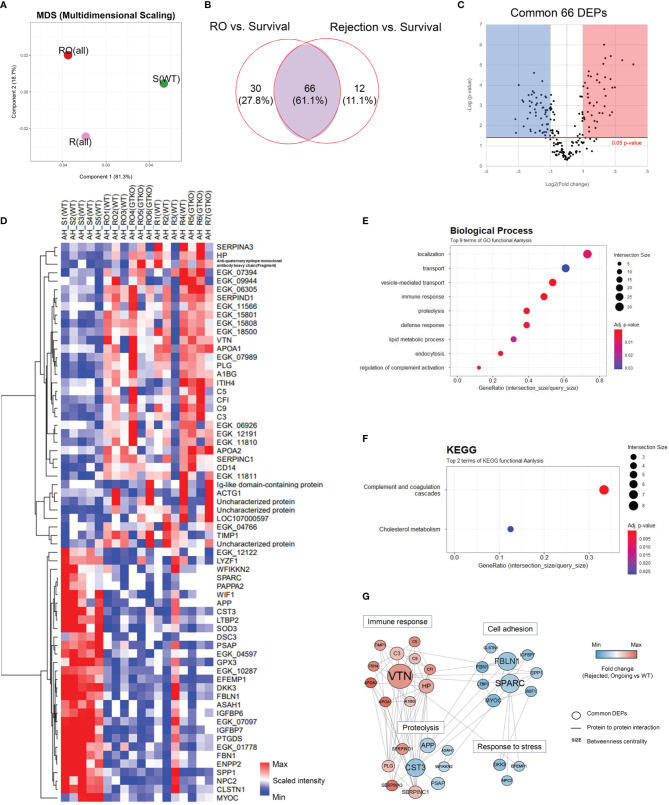
Proteomic analysis of the aqueous humor (AH). Proteins from a non-human primate library (*Macaca mulatta*) with at least 2-fold significant (*p* < 0.05) changes in the rejection ongoing (RO) and rejection (R) groups [compared with the survival (S) group] were analyzed. **(A)** Principal component analysis (PCA) of AH protein expression in the R, RO, and S groups. **(B)** Venn diagram depicting the comparison of differentially expressed proteins (DEPs) in the RO and R groups compared with the S group. A set of 66 proteins (common DEPs) exhibited a significant and more than 2-fold change in both the RO and R groups (compared with the S group). **(C)** A volcano plot showing the differential level of proteins in the RO and R groups compared with the S group. **(D)** Cluster heat map of the 66 common DEPs identified in AH. **(E, F)** Gene Ontology biological process classification **(E)** and Kyoto Encyclopedia of Genes and Genomes (KEGG) pathway enrichment analyses **(F)** of the common DEPs. Node size represents the gene ratio; node color represents the adjusted *p*-value. **(G)** Network modeling of the common DEPs in AH. A network model showing the biological processes affected, including the immune response, proteolysis, cell adhesion, and response to stress. The colors of the nodes represent proteins whose levels were greatly increased (red) or decreased (blue) in the RO and R groups compared with the S group. Large nodes indicate a high degree of connectivity (betweenness centrality) with other proteins in the model. Connections between nodes (gray lines) indicate either regulatory roles or physical interactions between proteins. WT, wild type; GTKO, α-1,3-galactosyltransferase gene-knockout.

**Figure 3:**
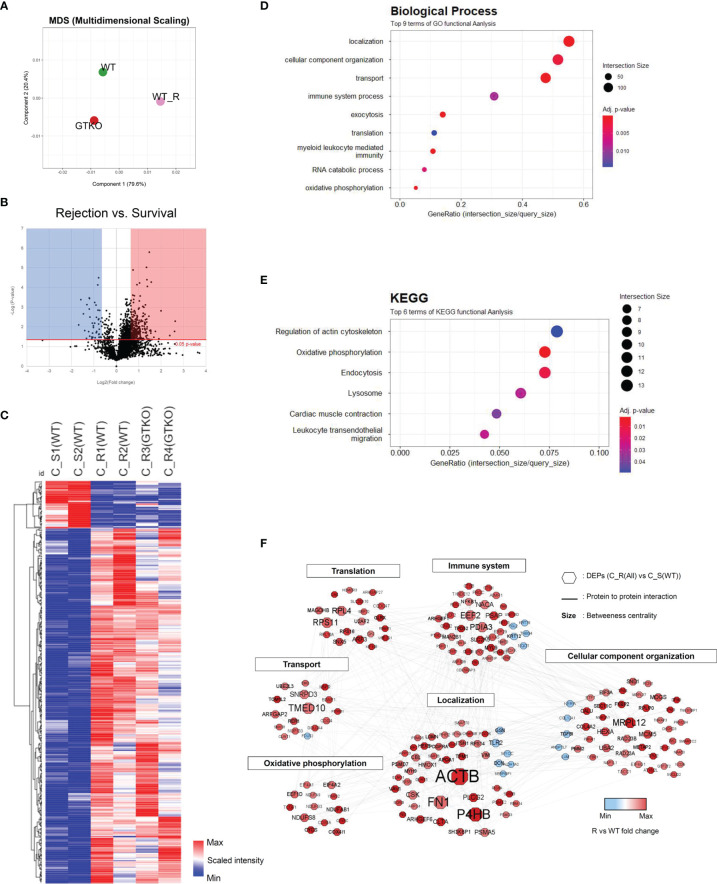
Proteomic analysis of the corneal tissues using a non-human primate (*Macaca mulatta*) library. Proteins with at least 1.5-fold significant (*p* < 0.05) changes in the rejection (R) groups [compared with the survival (S) group] were analyzed. **(A)** Principal component analysis of corneal tissue protein expression in the wild-type (WT) porcine cornea transplant group with rejection [C_R(WT)] or without rejection [survival; (C_S(WT)] and α-1,3‐galactosyltransferase gene‐knockout (GTKO) porcine cornea transplant group with rejection [C_R(GTKO)]. **(B)** A volcano plot showing the differential level of proteins in the R group compared with the S group. **(C)** Cluster heat map of DEPs identified in corneal tissue. **(D, E)** Gene Ontology biological process classification **(D)** and Kyoto Encyclopedia of Genes and Genomes (KEGG) pathway enrichment analyses **(E)** of the DEPs in corneal tissue. Node size represents the gene ratio; node color represents the adjusted *p*-value. **(F)** Network modeling of the DEPs in corneal tissue. The network model showing the biological processes affected, including the immune system, cellular component organization, and oxidative phosphorylation. The colors of the nodes represent proteins whose levels were greatly increased (red) or decreased (blue) in the R group compared with the S group. Large nodes indicate a high degree of connectivity (betweenness centrality) with other proteins in the model. Connections between nodes (gray lines) indicate either regulatory roles or physical interactions between proteins.

Quantitative proteomics analysis identified 649 host proteins in the AH samples. In the principal component analysis (PCA), the RO and rejection groups were close to each other and were distinctly different from the survival group (**Figure 2A**). Ninety-six and 78 proteins in the RO and rejection groups, respectively, were DEPs, which showed more than a 2-fold significant difference compared with those in the survival group. Sixty-six proteins were common DEPs in the RO and rejection groups (**Figures 2B, C**). A comprehensive list of these 66 DEPs was generated using a heat map (**Figure 2D**). To gain insight into the functional roles of DEPs associated with the progression of corneal xenograft rejection, we compared the GO of the biological processes (BPs) and KEGG pathway analysis using these 66 common DEPs. The most enriched BP pathways included localization, immune response, proteolysis, lipid metabolic processes, and complement activation regulation (**Figure 2E**). Among the DEPs of BP, proteins of C3, C5, C9, PLG, and VTN that are known to be involved in complement and coagulation cascades and APOA1 and APOA2 that are involved in cholesterol metabolism were upregulated. Proteins of prothrombin (EGK_06305), α-albumin (EGK_15801), and SERPINA3 were also upregulated. Enriched KEGG pathways were complement and coagulation cascades and cholesterol metabolism (**Figure 2F**). In KEGG, the upregulated DEPs were similar to those of BPs and were C3, C5, C9, PLG, VTN, SERPINC1, SERPIND1, PLG, VTN, APOA1, APOA2, and NPC2. To identify proteins that play a central role in corneal xenograft rejection, we used network models using DEPs from the AH and cornea using the STRING database (**Figure 2E**). Through this process, we could explore the potential logical cause–effect network connections to better understand the key proteins involved in crucial biological processes that may influence the mechanism of rejection in corneal xenotransplantation. In network models, DEPs were grouped into four modules, namely, immune response, cell adhesion, proteolysis, and response to stress, based on the GO BP and KEGG analyses (**Figure 3F**). Upregulated VTN, C3, C5, C9, and APOA1 formed a close network with immune response. In this model, VTN, SPARC, FBLN1, CST3, APP, HPP, and C3 possessed high betweenness centrality (**Figure 2G**).

### 3.2 Proteomics Analysis of the NHP Corneal Tissues in the Rejected and Survived Grafts

Quantitative proteomics analysis identified 3,062 host proteins in the corneal tissues. In PCA, the survival group showed a distinct difference from the rejection group (**Figure 3A**). Among the identified proteins, 416 proteins were chosen as DEPs of the cornea (fold change ≥ 1.5; *p* < 0.05) between the rejected and surviving grafts (**Figures 3B, C**). A heat map of the GO of the BPs during DEP enrichment analysis showed significantly represented GO BP terms (*p* < 0.05) for DEPs between the rejected and surviving groups. The most enriched BP pathways included localization, myeloid leukocyte-mediated immunity, immune system process, and oxidative phosphorylation (**Figure 3D**). Among the DEPs of BP, the proteins EEF2, GUSB, CTSH, CTSB, RNASE3, PNP, PTGES2, FES, CBL, UNC13D, HMOX1, LAMTOR1, NCF2, VIM, NF-κB1, PSMD7, PSMA5, and PSMD3, which are known to be involved in rejection-related immune responses, were upregulated. FGL2, which regulates immune-modulatory function, was downregulated. The proteins AIF1, STX8, PLCG2, TMED10, VAV1, ARHGAP27, FN1, ANXA5, CLTA, and M6PR in vesicle-mediated transport or localization of BPs were also upregulated. Enriched KEGG pathways included oxidative phosphorylation and lysosomes (**Figure 3E**). In KEGG, the upregulated DEPs were as follows: 1) NDUFS3, NDUFA13, NDUFAB1, NDUFA9, COX6C, COX4I1, COX5B, COX5A, ATP5PF, ATP5ME, ATP5PB, CYCS, and PDXK, which are related to electron transport chain (ETC) with immune responses; 2) CLTA, M6PR, and LGMN of lysosomal proteins to mediate an immune response; 3) NF-κB1 and proteasomes (PSMD7, PSMA5, and PSMD3); and 4) tropomycin 1 to 4 which may facilitate T-cell synaptic actin. In network models, DEPs were grouped into six modules, including immune system process, protein transport, oxidative phosphorylation, localization, and cellular component organization based on the GO BP and KEGG (**Figure 3F**) analysis. Upregulated NF-κB1, PSMD7, PSMA5, and PSMD3 in both BP and KEGG formed a close network with immune system processes within the protein–protein network model. Among the proteins that possessed the highest betweenness centrality (the top 10 protein–protein interactions), P4HB, TMED10, EEF2, and PDIA3 were enriched in the immune system process with high betweenness centrality in this network model.

### 3.3 Proteomics Analysis of the NHP Aqueous Humor and Corneal Tissues in the GTKO Rejected and WT Rejected Grafts

**Figure 4:**
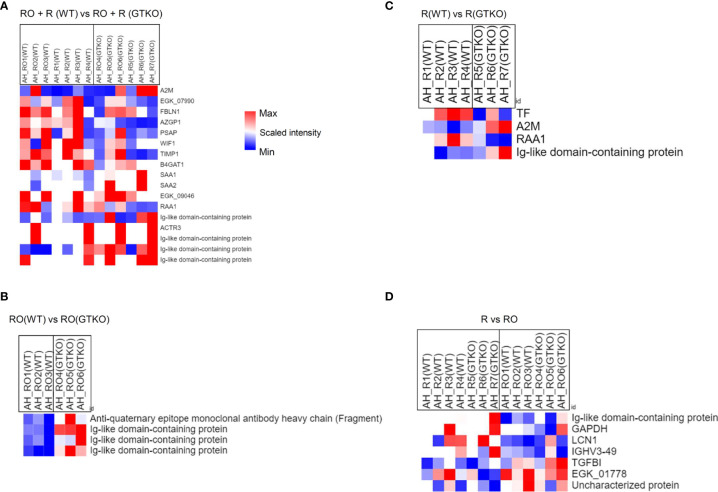
Proteomic analysis of the subgroups in aqueous humor (AH) using a non-human primate (*Macaca mulatta*) library. **(A)** Cluster heat map of differentially expressed proteins (DEPs) comparing subgroups transplanted with wild-type (WT) or α-1,3‐galactosyltransferase gene‐knockout (GTKO) porcine corneas when the rejection ongoing (RO) and rejection (R) groups were combined. **(B)** Cluster heat map of DEPs comparing subgroups transplanted with WT or GTKO porcine corneas in the RO group. **(C)** Cluster heat map of DEPs comparing subgroups transplanted with WT or GTKO porcine corneas in the R group. **(D)** Cluster heat map of DEPs comparing the R and RO groups regardless of donor corneal characteristics.

**Figure 5:**
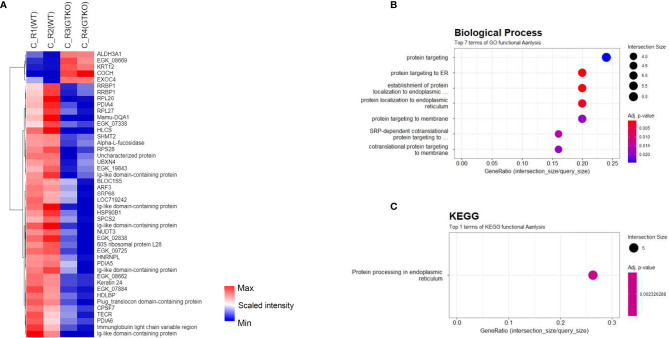
Proteomic analysis of the subgroups in corneal tissue. **(A)** Cluster heat map of differentially expressed proteins (DEPs) comparing subgroups transplanted with wild-type (WT) or α-1,3‐galactosyltransferase gene‐knockout (GTKO) porcine corneas in the rejection (R) group. **(B, C)** Gene Ontology biological process classification **(B)** and Kyoto Encyclopedia of Genes and Genomes (KEGG) pathway enrichment analyses **(C)** of the DEPs between subgroups transplanted with WT and GTKO porcine corneas in the R group. Node size represents the gene ratio; node color represents the adjusted *p*-value.

In the subgroup analysis, we evaluated whether the characteristics of the donor cornea (WT or GTKO) affected protein expression in the rejected cornea. After the RO and rejection groups were combined and AH was compared between the groups transplanted with WT and GTKO porcine corneas, 17 proteins showed a more than 2-fold significant difference, including AZGP1, Ig-like domain-containing protein, SAA1, SAA2, and RAA1 (**Figure 4A**). When the RO and rejection groups were analyzed separately (**Figures 4B, C**, respectively), there were a few DEPs between the groups transplanted with the WT and GTKO porcine corneas, and Ig-like domain-containing proteins were the common DEPs. When we compared the RO and rejection groups, seven proteins showed greater than 2-fold significant difference (**Figure 4D**). Among the proteins in the rejection group, four proteins, including lipocalin 1 (LCN1) and immunoglobulin heavy variable 3-49 (IGHV3-49), were upregulated, and three proteins, including transforming growth factor-beta-induced protein (TGFBI), were downregulated. In the subgroup analysis of AH, there was no enriched GO BP or KEGG pathway because of the small number of DEPs. In the cornea, quantitative proteome analysis revealed a total of 45 DEPs between GTKO rejected and WT rejected grafts (**Figure 5A**). Most BPs included protein targeting and KEGG enriched protein processing in the endoplasmic reticulum (**Figures 5B, C**). Among the DEPs of BP, the proteins SRP68, RPL26, SPCS2, RPS28, and RPL27 were downregulated, whereas the protein EXOC4 was upregulated. Among the DEPs of KEGG, all the proteins HSP90B1, RRBP1, PDIA4, PDIA6, and UBXN4 were down-regulated.

### 3.4 Validation of Biomarker Candidates

**Figure 6:**
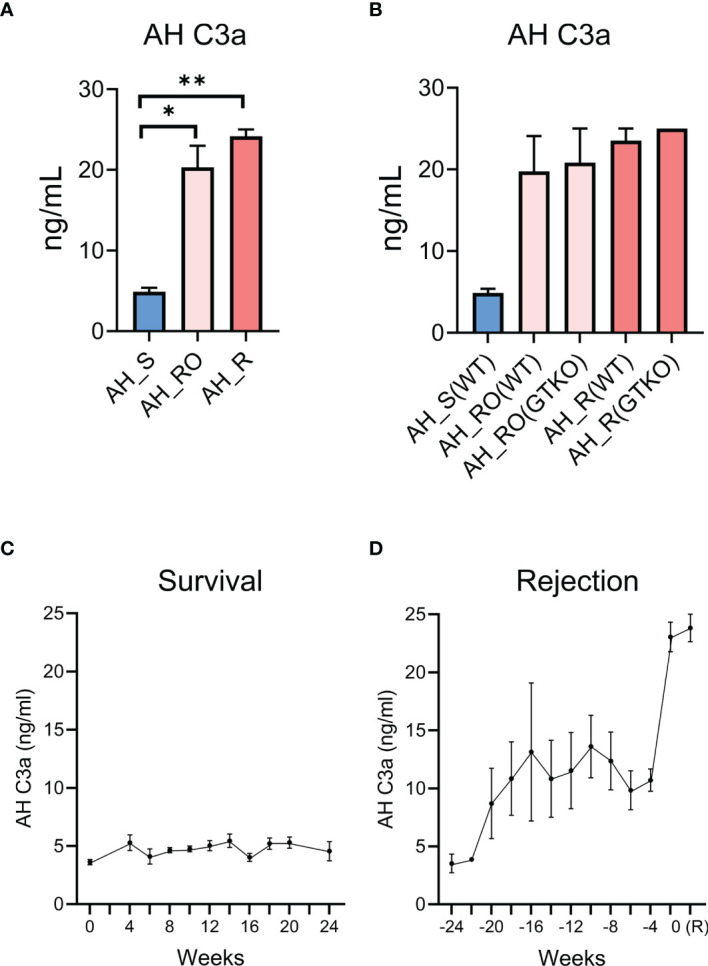
Validation of aqueous humor (AH) C3a as a putative biomarker for corneal xenograft rejection. **(A)** Concentration of aqueous humor (AH) C3a in the survival (AH_S), rejection ongoing (AH_RO), and rejection (AH_R) groups. The concentration of AH C3a was significantly higher in the rejection ongoing (RO) and rejection (R) groups compared with that in the survival (S) group (*p* = 0.034 and *p* = 0.002; Kruskal–Wallis test with Dunn’s multiple comparisons test). **(B)** Subgroup analysis of the concentration of AH C3a. AH_RO(WT) and AH_RO(GTKO) mean the AH of the RO subgroup transplanted with wild type (WT) and α-1,3‐galactosyltransferase gene‐knockout (GTKO) porcine cornea, respectively. AH_R(WT) and AH_R(GTKO) mean the AH of the R subgroup transplanted with WT and GTKO porcine cornea, respectively. The concentration did not differ according to the type of donor cornea (WT versus GTKO). **(C)** Time-dependent changes of AH C3a concentration in the survival group after xenotransplantation. The time point of xenotransplantation was set as the reference time point (0 week). **(D)** Time-dependent changes of AH C3a concentration in the rejection group after xenotransplantation. The time point at which xenograft rejection was set as the reference time point (0 week) and the AH C3a concentrations before rejection were shown. R, rejection. Data are presented as means ± standard error. *p < 0.05, **p < 0.01.

In the AH, the complement process was found to be related to graft rejection. As C3 activation is commonly involved in complement pathways, we measured the complement activation product C3a by using ELISA. The concentration was significantly higher in the RO and rejection groups than in the survival group (**Figure 6A**). However, the concentration was not significantly different according to the type of donor cornea (WT versus GTKO) (**Figure 6B**). When the concentration of AH C3a in each recipient was analyzed in a time-dependent manner, there was no significant change in the survival group (**Figure 6C**). Meanwhile, the concentration of AH C3a increased over time before rejection (**Figure 6D**).

## 4 Discussion

In this study, we investigated proteomic biomarkers and their associated processes in AH and corneal tissue after pig-to-NHP corneal xenotransplantation. This is the first proteomic study to identify biomarkers related to graft rejection after corneal xenotransplantation, while only one study conducted proteomic analyses of AH after corneal allotransplantation (22). We used a combination of LFQ and TMT quantitative proteomics analyses. We isolated and identified 649 and 3,062 proteins from the aqueous humor and corneal tissues, respectively. Among them, 66 DEPs from aqueous humors and 416 DEPs from corneal tissues were associated with xenogeneic rejection. The most important proteins in the aqueous humor were complement proteins (C3, C5, and C9) and cholesterol metabolic proteins (APOA1 and APOA2). In the corneal tissue, NFKB1 and proteasome-related proteins (PSMD7, PSMA5, and PMDB) were found to be critical for the processing of class I MHC peptides. Corneal proteins involved in oxidative phosphorylation-related pathways and leukocyte activation-related pathways were also found to be involved in graft rejection.

Several studies have reported that activation of the complement pathway in AH after corneal xenotransplantation is associated with corneal xenograft rejection (14, 23, 24). In line with previous findings, our proteomic analysis showed that the complement proteins C3, C5, and C9 and complement activation inhibitor vitronectin (VTN) were increased in the RO and rejection groups than in the survival group. However, C2 and C4 levels did not change. It has not been elucidated which complement activation pathways are important, and the results suggest that alternative pathways, rather than classical or lectin pathways, are mainly involved in corneal xenogeneic rejection (25). Interestingly, when comparing AH in the RO group transplanted with GTKO porcine cornea with that in the survival group, six proteins (C3, C5, C7, C9, C8B, and C8G) were enriched in the “Coronavirus disease - COVID-19” KEGG pathway (data not shown). Coronavirus infection results in the activation of multiple complement pathways (26). Clinically, there have been reports of corneal allograft rejection after COVID-19 vaccination (27–29). Based on the results of our study, an increase in the complement of aqueous humor may be related to the rejection; future research will be necessary in this regard.

The levels of APOA1 and APOA2, which are critical components involved in the formation of high-density lipoprotein (HDL) (30), were increased in the AH of the RO and rejection groups. Similar to our study, APOA1 and APOA2 levels were reportedly elevated in AH in patients with glaucoma shunt devices and corneal endothelial damage (31). However, in a study evaluating heterotopic islet allotransplantation in the AH, APOA1 levels decreased in AH during the progression of rejection (7). Lower levels of plasma APOA1 are associated with acute renal allograft rejection (32), whereas higher APOA1 levels inhibit the rejection of cardiac transplants due to its anti-inflammatory and antioxidant properties (33). The relationship between APOA1 or APOA2 levels and graft rejection may depend on the organ type (allotransplant or xenotransplant) and the sample type (AH or plasma). Anti-inflammatory HDL can turn proinflammatory during the acute phase and under inflammatory conditions (34). Considering the anti-inflammatory properties of HDL cholesterol, elevation in APOA1 and APOA2 levels in AH observed in this study may be secondary to increased inflammation. Taken together, increased levels of APOA1 and APOA2, which are involved in the cholesterol pathway in AH, can be used as biomarkers of corneal xenotransplant rejection; however, further evaluation is necessary to elucidate their precise role.

Increased levels of other proteins, such as the acute phase response protein SERPINA3 as well as α-albumin, a protein related to the inflammatory breakdown of the blood–aqueous barrier, were observed (EGK_15801) (22, 35). These results correspond with those of a previous study that performed proteomic analysis of AH in patients with acute rejection after corneal allotransplantation and showed that the levels of albumin and serine proteinase inhibitors were significantly increased in the AH of rejected corneal allografts (22).

Comparing the results of xenografts with those of allografts, complement or cholesterol-related proteins did not change in aqueous humor of cases with allograft rejection (22), indicating that these proteins are more likely to influence corneal xenograft rejection than allograft rejection.

Nuclear factor-κB (NF-κB), proteasomes, protein disulfide-isomerase, and immunometabolism regulated by ETC coupled with oxidative phosphorylation appear to play important roles in corneal xenogeneic rejection under treatment with conventional immunosuppressive agents. NF-κB signaling contributes to the activation of T or B cells or hampers regulatory T-cell (Treg) activation in allogeneic as well as xenogeneic rejection (36–43). NF-κB signaling can be activated by various signals, including those mediated *via* Toll-like receptors (TLRs), tumor necrosis factor receptor (TNFR), T-cell receptor (TCR), B-cell receptor (BCR), or membrane attack complex (MAC) of the complement (37, 42). Given that NF-κB signaling is involved in both allogeneic and xenogeneic rejection (38, 39), complement activation can trigger the NF-κB pathway *via* MAC to induce corneal xenogeneic rejection. In addition, NF-κB translocates to the nucleus following IκB degradation to induce activation of the immune cascade (44, 45). Proteasomes of which the PSMD family genes are positively correlated with ubiquinone metabolism, the immune system, and cell-cycle regulatory pathways are also related to NF-κB activation (44–46). Considering that proteasome 26S non-ATPase subunits, such as PSMD 3 and PSMD 7, are positively correlated with the progression of various cancers *via* NF-κB activation or immune cell infiltration (47–49), proteasomes may be related to xenogeneic rejection *via* close interactions with NF-κB1. MHC I-processing-related proteins such as Rho GTPase-activating protein 45 (ARHGAP45) and protein disulfide-isomerase (P4HB, PDIA3) are involved in xenogeneic rejection, with a high betweenness centrality (50, 51). Metabolism of T cells or macrophages through ETCs linking oxidative phosphorylation is closely related to alloimmune responses (52–54). Proteins such as “vesicle-mediated transport,” allograft inflammatory factor 1 (AIF1), clathrin light chain (CLTA), mannose-6-phosphate receptor (M6PR), PLCG2, STX8, and VAV1 are related to immune responses such as allograft rejection, B-cell maturation, lytic granule trafficking in cytotoxic T cells, and TCR-induced integrin clustering (12, 55–59). Notably, clathrin-mediated endocytosis is enriched in corneal allograft rejection (12). In addition, the S100 protein, seen in corneal inflammation as a regulator of macrophage inflammation (60–63), was upregulated. Indeed, S100A9 and S100A8 expression levels were also significantly elevated in rejected grafts compared with those in normal corneas (**Supplementary Figure 1**). Given that it has not yet been elucidated which biological pathways are mainly enriched in xenogeneic rejection, this study gives us informative evidence on protein–protein interactions in corneal xenogeneic rejection. Inhibition of NF-κB activation and proteasome subunits, along with steroids and tacrolimus, may enable the reduction of the incidence of xenogeneic rejection.

In the subgroup AH analysis, we compared the groups transplanted with WT and GTKO porcine corneas. Surprisingly, there were only a few DEPs in either the AH or rejected grafts. Most DEPs in the AH were Ig-like domain-containing proteins. When the GTKO cornea was transplanted, it was expected that the antibody reactions would be mild; however, the expression of Ig-like domain-containing protein was higher in the GTKO group than in the WT group. The GTKO porcine cornea showed decreased αGal antigen expression; however, it may increase the expression levels of non-Gal antigens. Indeed, elevated levels of non-Gal IgG were reported in rejected GTKO porcine corneas after corneal xenotransplantation (5). In addition, anti-CD20 antibodies (rituximab) were not used in the GTKO porcine cornea-transplanted group; this may have influenced the results. In the subgroup corneal analysis, proteins related to platelet activation or thrombosis were downregulated in GTKO corneas, compared with those in the case of WT corneas. Nevertheless, both GTKO and WT grafts were rejected similarly. This suggests that the other biological pathways mentioned above are still present in both groups.

Because the cornea is an avascular organ, the effect of hyperacute rejection involving alpha Gal is expected to be less than that of a solid organ such as the kidney and heart. In addition, GTKO porcine cornea may increase the expression levels of non-Gal antigens because the level of non-Gal antigens from GTKO porcine fibroblasts is much higher than that from WT porcine fibroblasts (64). Indeed, elevated levels of non-Gal IgG were reported in rejected GTKO porcine corneas after corneal xenotransplantation. These reasons are supposed to be the reason why GTKO was not effective in pig-to-non-human primate corneal xenotransplantation.

There is only one study regarding pig-to-NHP solid organ xenotransplantation. In the study of heart xenotransplantation, 14 potential proteins including fibronectin, annexin, and vimentin were identified as targets of IgG antibodies (8). In line with the study, fibronectin (EGK_04766) was a common DEP in AH, and fibronectin (FN1), annexin (ANXA5), and vimentin (VIM) were DEPs in corneal tissue, and those proteins were higher in both the rejection and rejection ongoing groups than in the survival group. Especially, fibronectin is regarded as the predominant xeno-antigen in GTKO porcine tissue (65). Although fibronectin was not different between the GTKO rejected and WT rejected grafts, it may be a candidate target protein to reduce rejection in both pig-to-NHP corneal and solid organ xenotransplantation.

This study has several limitations. First, the immunosuppressive regimen was variable among the subjects, which may have affected protein expression. There were no different basic conditions of the recipients with and without immunosuppression, and we performed several NHP studies to find the most appropriate immunosuppressive regimen. Through the studies of pig-to-NHP corneal xenotransplantation using various immunosuppressant regimens (5, 16), we found rituximab as one of the key drugs to reduce xenogeneic rejection. Therefore, most recipients with survived graft were immunosuppressed under the regimen that included rituximab. However, this study revealed the difference between the groups that showed long-term graft survival and rejection, even under immunosuppression. Therefore, it is necessary to administer drugs that target DEPs that are associated with rejection. Second, the sample size was small. Because corneal xenotransplantation was performed on NHPs, a large number of samples could not be collected. Further studies are needed to confirm these results. Third, because the AH sample is composed mostly of water and extracellular proteins, it is difficult to identify more than 1,000 proteins in the samples. In addition, the low protein concentration in the AH sample restricted the protein identification process. Moreover, the database of *M. mulatta* and *S. scrofa* lacks reviewed data, unlike human or mouse databases. These sample- and species-associated limitations further restricted the protein identification process. In addition, the cornea is composed of abundant amounts of ECM and collagen matrix, and the major proteins present in the cornea are mostly isoforms of collagen or ECM proteins. Of course, due to the nature of the cornea, mass spectrometry analysis with a higher resolution and novel methods for analyzing more proteins, such as cytokines or interleukins, will be necessary in order to deepen our understanding of the cornea. Due to the lack of samples, it was difficult to quantify each protein using targeted methods to validate our results.

In this study, we aimed to evaluate the proteomic changes in the corneal tissue, identify AH biomarkers related to graft rejection, and explore the changes in xenogeneic rejection-networking pathways according to donor characteristics (WT and GTKO porcine graft) after corneal xenotransplantation. The complement pathways of the AH can be used as predictive biomarkers in xenogeneic rejection, and alternative pathways are expected to be mainly involved in the complement system. The difference in protein expression according to the donor characteristics was small. Proteins related to platelet activation or thrombosis were downregulated in the GTKO cornea (compared with their expression in WT). Nevertheless, both the GT KO and WT grafts were similarly rejected, suggesting that the cornea was affected by other biological pathways related to rejection even when the GTKO cornea was used. Upon combining the results of proteomic analysis of the AH and cornea, the complement pathway of AH or corneal NFKB/proteasome-related proteins was found to be associated with graft rejection. It is presumed that the combination of immunosuppressive agents used in the study is not sufficient to inhibit these pathways and the additional use of eculizumab (a complement pathway inhibitor) or bortezomib (a proteasome/NF-κB activation inhibitor) can reduce xenogeneic rejection after corneal xenotransplantation. In addition, the use of genetically modified pigs expressing GTKO, CD46, CD55, and/or human A20 that modulate the complement and NF-κB pathway may also reduce rejection in pig-to-non-human primate corneal xenotransplantation.

In conclusion, the NFKB/proteasome-related proteins were associated with corneal xenograft rejection even with immunosuppressive agents. Furthermore, the complement activation product C3a was found to be an aqueous biomarker for the progression of xenogeneic rejection in pig-to-NHP corneal xenotransplantation, and xenogeneic rejection-networking pathways were found to be not so different between WT and GTKO corneal grafted NHPs. Further studies with a larger number of experiments are needed to determine and select more accurate markers and to validate our hypotheses.

## Data Availability Statement

The mass spectrometry proteomics data have been deposited to the ProteomeXchange Consortium *via* the PRIDE partner repository with the dataset identifier PXD031320.

## Ethics Statement

The studies involving human participants were reviewed and approved by Seoul National University Hospital Institutional Review Board for the use of human corneas (No. 1102-092-353). The patients/participants provided their written informed consent to participate in this study. The animal study was reviewed and approved by Seoul National University (SNU) (IACUC: SNU‐151102‐3) and SNU Hospital (IACUC: 15‐0171, 18-0160).

## Author Contributions

MK and KK conceived and designed this study. JO, CY, and JR performed the experiments. JO, CY, KK, and MK analyzed and interpreted the data and prepared the manuscript. All authors contributed to the article and approved the submitted version.

## Funding

This research was carried out with the support from the Korea Healthcare Technology R&D Project, Ministry for Health & Welfare, Republic of Korea (grant number: HI13C0954), and the Gyeonggi-do Regional Research Center Program of Gyeonggi Province [grant number: GRRC-Kyung Hee 2018(B03)].

## Conflict of Interest

The authors declare that the research was conducted in the absence of any commercial or financial relationships that could be construed as a potential conflict of interest.

## Publisher’s Note

All claims expressed in this article are solely those of the authors and do not necessarily represent those of their affiliated organizations, or those of the publisher, the editors and the reviewers. Any product that may be evaluated in this article, or claim that may be made by its manufacturer, is not guaranteed or endorsed by the publisher.

## References

[1] Tan DT Dart JK Holland EJ Kinoshita S. Corneal Transplantation. Lancet (2012) 379(9827):1749–61. doi: 10.1016/s0140-6736(12)60437-1 22559901

[2] Gain P Jullienne R He Z Aldossary M Acquart S Cognasse F. Global Survey of Corneal Transplantation and Eye Banking. JAMA Ophthalmol (2016) 134(2):167–73. doi: 10.1001/jamaophthalmol.2015.4776 26633035

[3] Yoon CH Choi HJ Kim MK. Corneal Xenotransplantation: Where Are We Standing? Prog Retin Eye Res (2021) 80:100876. doi: 10.1016/j.preteyeres.2020.100876 32755676 PMC7396149

[4] Lai L Kolber-Simonds D Park KW Cheong HT Greenstein JL Im GS. Production of Alpha-1,3-Galactosyltransferase Knockout Pigs by Nuclear Transfer Cloning. Science (2002) 295(5557):1089–92. doi: 10.1126/science.1068228 11778012

[5] Yoon CH Choi SH Choi HJ Lee HJ Kang HJ Kim JM. Long-Term Survival of Full-Thickness Corneal Xenografts From A1,3-Galactosyltransferase Gene-Knockout Miniature Pigs in Non-Human Primates. Xenotransplantation (2020) 27(1):e12559. doi: 10.1111/xen.12559 31566261

[6] Chu X Zhang B Koeken V Gupta MK Li Y. Multi-Omics Approaches in Immunological Research. Front Immunol (2021) 12:668045. doi: 10.3389/fimmu.2021.668045 34177908 PMC8226116

[7] Alcazar O Hernandez LF Nakayasu ES Piehowski PD Ansong C Abdulreda MH. Longitudinal Proteomics Analysis in the Immediate Microenvironment of Islet Allografts During Progression of Rejection. J Proteomics (2020) 223:103826. doi: 10.1016/j.jprot.2020.103826 32442648 PMC7483621

[8] Byrne GW Stalboerger PG Davila E Heppelmann CJ Gazi MH McGregor HC. Proteomic Identification of Non-Gal Antibody Targets After Pig-To-Primate Cardiac Xenotransplantation. Xenotransplantation (2008) 15(4):268–76. doi: 10.1111/j.1399-3089.2008.00480.x PMC2586876 18957049

[9] Subbannayya Y Pinto SM Mohanty V Dagamajalu S Prasad TSK Murthy KR. What Makes Cornea Immunologically Unique and Privileged? Mechanistic Clues From a High-Resolution Proteomic Landscape of the Human Cornea. Omics (2020) 24(3):129–39. doi: 10.1089/omi.2019.0190 32125911

[10] Yamaguchi T Higa K Tsubota K Shimazaki J. Elevation of Preoperative Recipient Aqueous Cytokine Levels in Eyes With Primary Graft Failure After Corneal Transplantation. Mol Vis (2018) 24:613–20. PMC6139874 30262982

[11] Flynn TH Mitchison NA Ono SJ Larkin DF. Aqueous Humor Alloreactive Cell Phenotypes, Cytokines and Chemokines in Corneal Allograft Rejection. Am J Transplant (2008) 8(7):1537–43. doi: 10.1111/j.1600-6143.2008.02285.x 18557741

[12] Huang F Xu J Jin H Tan J Zhang C. Itraq-Based Quantitative Proteomic Analysis of Tear Fluid in a Rat Penetrating Keratoplasty Model With Acute Corneal Allograft Rejection. Invest Ophthalmol Vis Sci (2015) 56(6):4117–24. doi: 10.1167/iovs.14-16207 26114489

[13] Hossain P. Biomarkers for Corneal Graft Rejection? Eye (Lond) (2009) 23(2):247. doi: 10.1038/eye.2008.308 19214163

[14] Yoon CH Choi SH Lee HJ Kang HJ Kim MK. Predictive Biomarkers for Graft Rejection in Pig-To-Non-Human Primate Corneal Xenotransplantation. Xenotransplantation (2019) 26(4):e12515. doi: 10.1111/xen.12515 30983050 PMC6717035

[15] Choi SH Yoon CH Lee HJ Kim HP Kim JM Che JH. Long-Term Safety Outcome of Systemic Immunosuppression in Pig-To-Nonhuman Primate Corneal Xenotransplantation. Xenotransplantation (2018) 25(4):e12442. doi: 10.1111/xen.12442 30264877 PMC6166667

[16] Kim J Choi SH Lee HJ Kim HP Kang HJ Kim JM. Comparative Efficacy of Anti-Cd40 Antibody-Mediated Costimulation Blockade on Long-Term Survival of Full-Thickness Porcine Corneal Grafts in Nonhuman Primates. Am J Transplant (2018) 18(9):2330–41. doi: 10.1111/ajt.14913 29722120

[17] Cox J Mann M. Maxquant Enables High Peptide Identification Rates, Individualized P.P.B.-Range Mass Accuracies and Proteome-Wide Protein Quantification. Nat Biotechnol (2008) 26(12):1367–72. doi: 10.1038/nbt.1511 19029910

[18] Wisniewski JR Zougman A Nagaraj N Mann M. Universal Sample Preparation Method for Proteome Analysis. Nat Methods (2009) 6(5):359–62. doi: 10.1038/nmeth.1322 19377485

[19] Thompson A Schafer J Kuhn K Kienle S Schwarz J Schmidt G. Tandem Mass Tags: A Novel Quantification Strategy for Comparative Analysis of Complex Protein Mixtures by Ms/Ms. Anal Chem (2003) 75(8):1895–904. doi: 10.1021/ac0262560 12713048

[20] Raudvere U Kolberg L Kuzmin I Arak T Adler P Peterson H. G:Profiler: A Web Server for Functional Enrichment Analysis and Conversions of Gene Lists (2019 Update). Nucleic Acids Res (2019) 47(W1):W191–8. doi: 10.1093/nar/gkz369 PMC6602461 31066453

[21] Szklarczyk D Gable AL Nastou KC Lyon D Kirsch R Pyysalo S. The String Database in 2021: Customizable Protein-Protein Networks, and Functional Characterization of User-Uploaded Gene/Measurement Sets. Nucleic Acids Res (2021) 49(D1):D605–D12. doi: 10.1093/nar/gkaa1074 PMC7779004 33237311

[22] Funding M Vorum H Honoré B Nexø E Ehlers N. Proteomic Analysis of Aqueous Humour From Patients With Acute Corneal Rejection. Acta Ophthalmol Scand (2005) 83(1):31–9. doi: 10.1111/j.1600-0420.2005.00381.x 15715554

[23] Larkin DF Williams KA. The Host Response in Experimental Corneal Xenotransplantation. Eye (Lond) (1995) 9( Pt 2):254–60. doi: 10.1038/eye.1995.49 7556727

[24] Choi HJ Kim MK Lee HJ Ko JH Jeong SH Lee JI. Efficacy of Pig-To-Rhesus Lamellar Corneal Xenotransplantation. Invest Ophthalmol Vis Sci (2011) 52(9):6643–50. doi: 10.1167/iovs.11-7273 21743020

[25] Thurman JM Holers VM. The Central Role of the Alternative Complement Pathway in Human Disease. J Immunol (2006) 176(3):1305–10. doi: 10.4049/jimmunol.176.3.1305 16424154

[26] Java A Apicelli AJ Liszewski MK Coler-Reilly A Atkinson JP Kim AH. The Complement System in Covid-19: Friend and Foe? JCI Insight (2020) 5(15):e140711. doi: 10.1172/jci.insight.140711 PMC7455060 32554923

[27] Phylactou M Li JO Larkin DFP. Characteristics of Endothelial Corneal Transplant Rejection Following Immunisation With Sars-Cov-2 Messenger Rna Vaccine. Br J Ophthalmol (2021) 105(7):893–6. doi: 10.1136/bjophthalmol-2021-319338 33910885

[28] Rallis KI Ting DSJ Said DG Dua HS. Corneal Graft Rejection Following Covid-19 Vaccine. Eye (Lond) (2021). doi: 10.1038/s41433-021-01671-2 PMC8380858 34426655

[29] Nioi M d'Aloja E Fossarello M Napoli PE. Dual Corneal-Graft Rejection After Mrna Vaccine (Bnt162b2) for Covid-19 During the First Six Months of Follow-Up: Case Report, State of the Art and Ethical Concerns. Vaccines (Basel) (2021) 9(11):1274. doi: 10.3390/vaccines9111274 34835205 PMC8620000

[30] Millán J Pintó X Muñoz A Zúñiga M Rubiés-Prat J Pallardo LF. Lipoprotein Ratios: Physiological Significance and Clinical Usefulness in Cardiovascular Prevention. Vasc Health Risk Manag (2009) 5:757–65. doi: 10.2147/VHRM.S6269 PMC2747394 19774217

[31] Anshu A Price MO Richardson MR Segu ZM Lai X Yoder MC. Alterations in the Aqueous Humor Proteome in Patients With a Glaucoma Shunt Device. Mol Vis (2011) 17:1891–900. PMC3144728 21850163

[32] Ziegler ME Chen T LeBlanc JF Wei X Gjertson DW Li KC. Apolipoprotein A1 and C-Terminal Fragment of A-1 Antichymotrypsin Are Candidate Plasma Biomarkers Associated With Acute Renal Allograft Rejection. Transplantation (2011) 92(4):388–95. doi: 10.1097/TP.0b013e318225db6a PMC3878300 21730889

[33] Hsieh GR Schnickel GT Garcia C Shefizadeh A Fishbein MC Ardehali A. Inflammation/Oxidation in Chronic Rejection: Apolipoprotein a-I Mimetic Peptide Reduces Chronic Rejection of Transplanted Hearts. Transplantation (2007) 84(2):238–43. doi: 10.1097/01.tp.0000268509.60200.ea 17667816

[34] Yu R Yekta B Vakili L Gharavi N Navab M Marelli D. Proatherogenic High-Density Lipoprotein, Vascular Inflammation, and Mimetic Peptides. Curr Atheroscler Rep (2008) 10(2):171–6. doi: 10.1007/s11883-008-0025-z 18417073

[35] Pescosolido N Barbato A Pascarella A Giannotti R Genzano M Nebbioso M. Role of Protease-Inhibitors in Ocular Diseases. Molecules (2014) 19(12):20557–69. doi: 10.3390/molecules191220557 PMC6271012 25493637

[36] Blanchett S Boal-Carvalho I Layzell S Seddon B. Nf-Kb and Extrinsic Cell Death Pathways - Entwined Do-Or-Die Decisions for T Cells. Trends Immunol (2021) 42(1):76–88. doi: 10.1016/j.it.2020.10.013 33246882

[37] Xie CB Jane-Wit D Pober JS. Complement Membrane Attack Complex: New Roles, Mechanisms of Action, and Therapeutic Targets. Am J Pathol (2020) 190(6):1138–50. doi: 10.1016/j.ajpath.2020.02.006 PMC7280757 32194049

[38] Bian J Wang T Sun J He X Wu Z Zhang S. Targeting Nf-Kb C-Rel in Regulatory T Cells to Treat Corneal Transplantation Rejection. Am J Transplant (2021) 21(12):3858–70. doi: 10.1111/ajt.16760 34254428

[39] Shen Z Ye W Ten X. Suppression of Nf-Kappab P65 Expression Attenuates Delayed Xenograft Rejection. Xenotransplantation (2013) 20(2):123–30. doi: 10.1111/xen.12027 23489828

[40] Suchanek O Clatworthy MR. Novel Strategies to Target the Humoral Alloimmune Response. Hla (2020) 96(6):667–80. doi: 10.1111/tan.14092 33022883

[41] Lunsford KE Barbas AS Brennan TV. Recent Advances in Immunosuppressive Therapy for Prevention of Renal Allograft Rejection. Curr Opin Organ Transplant (2011) 16(4):390–7. doi: 10.1097/MOT.0b013e328348b420 21666473

[42] Yang MG Sun L Han J Zheng C Liang H Zhu J. Biological Characteristics of Transcription Factor Relb in Different Immune Cell Types: Implications for the Treatment of Multiple Sclerosis. Mol Brain (2019) 12(1):115. doi: 10.1186/s13041-019-0532-6 31881915 PMC6935142

[43] Ma Y Xie B Guo J Chen Y Zhong M Lin Q. Leflunomide Inhibits Rat-To-Mouse Cardiac Xenograft Rejection by Suppressing Adaptive Immune Cell Response and Nf-Kb Signaling Activation. Cell Transplant (2021) 30:9636897211054503. doi: 10.1177/09636897211054503 34814739 PMC8647224

[44] Eskandari SK Seelen MAJ Lin G Azzi JR. The Immunoproteasome: An Old Player With a Novel and Emerging Role in Alloimmunity. Am J Transplant (2017) 17(12):3033–9. doi: 10.1111/ajt.14435 28719024

[45] Grisham MB Palombella VJ Elliott PJ Conner EM Brand S Wong HL. Inhibition of Nf-Kappa B Activation in Vitro and in Vivo: Role of 26s Proteasome. Methods Enzymol (1999) 300:345–63. doi: 10.1016/s0076-6879(99)00140-8 9919536

[46] Tanaka K. The Proteasome: Overview of Structure and Functions. Proc Jpn Acad Ser B Phys Biol Sci (2009) 85(1):12–36. doi: 10.2183/pjab.85.12 PMC3524306 19145068

[47] Bencomo-Alvarez AE Rubio AJ Olivas IM Gonzalez MA Ellwood R Fiol CR. Proteasome 26s Subunit, Non-Atpases 1 (Psmd1) and 3 (Psmd3), Play an Oncogenic Role in Chronic Myeloid Leukemia by Stabilizing Nuclear Factor-Kappa B. Oncogene (2021) 40(15):2697–710. doi: 10.1038/s41388-021-01732-6 PMC7952820 33712704

[48] Xuan DTM Wu CC Kao TJ Ta HDK Anuraga G Andriani V. Prognostic and Immune Infiltration Signatures of Proteasome 26s Subunit, Non-Atpase (Psmd) Family Genes in Breast Cancer Patients. Aging (Albany NY) (2021) 13(22):24882–913. doi: 10.18632/aging.203722 PMC8660617 34839279

[49] Zhang S Yu S Wang J Cheng Z. Identification of Psmd7 as a Prognostic Factor Correlated With Immune Infiltration in Head and Neck Squamous Cell Carcinoma. Biosci Rep (2021) 41(3):BSR20203829. doi: 10.1042/bsr20203829 33687056 PMC7990087

[50] Cho K Cho S Lee SO Oh C Kang K Ryoo J. Redox-Regulated Peptide Transfer From the Transporter Associated With Antigen Processing to Major Histocompatibility Complex Class I Molecules by Protein Disulfide Isomerase. Antioxid Redox Signal (2011) 15(3):621–33. doi: 10.1089/ars.2010.3756 21299467

[51] Lepin EJ Jin YP Barwe SP Rozengurt E Reed EF. Hla Class I Signal Transduction Is Dependent on Rho Gtpase and Rok. Biochem Biophys Res Commun (2004) 323(1):213–7. doi: 10.1016/j.bbrc.2004.08.082 15351723

[52] Yin M O'Neill LAJ. The Role of the Electron Transport Chain in Immunity. FASEB J (2021) 35(12):e21974. doi: 10.1096/fj.202101161R 34793601

[53] Priyadharshini B Turka LA. T-Cell Energy Metabolism as a Controller of Cell Fate in Transplantation. Curr Opin Organ Transplant (2015) 20(1):21–8. doi: 10.1097/mot.0000000000000149 PMC4387772 25563988

[54] Allocco JB Alegre ML. Exploiting Immunometabolism and T Cell Function for Solid Organ Transplantation. Cell Immunol (2020) 351:104068. doi: 10.1016/j.cellimm.2020.104068 32139072 PMC7150626

[55] Sikora M Kopeć B Piotrowska K Pawlik A. Role of Allograft Inflammatory Factor-1 in Pathogenesis of Diseases. Immunol Lett (2020) 218:1–4. doi: 10.1016/j.imlet.2019.12.002 31830499

[56] Jackson JT Mulazzani E Nutt SL Masters SL. The Role of Plcγ2 in Immunological Disorders, Cancer, and Neurodegeneration. J Biol Chem (2021) 297(2):100905. doi: 10.1016/j.jbc.2021.100905 34157287 PMC8318911

[57] Krawczyk C Oliveira-dos-Santos A Sasaki T Griffiths E Ohashi PS Snapper S. Vav1 Controls Integrin Clustering and Mhc/Peptide-Specific Cell Adhesion to Antigen-Presenting Cells. Immunity (2002) 16(3):331–43. doi: 10.1016/s1074-7613(02)00291-1 11911819

[58] Bhat SS Friedmann KS Knörck A Hoxha C Leidinger P Backes C. Syntaxin 8 Is Required for Efficient Lytic Granule Trafficking in Cytotoxic T Lymphocytes. Biochim Biophys Acta (2016) 1863(7 Pt A):1653–64. doi: 10.1016/j.bbamcr.2016.04.014 27094127

[59] Dressel R von Figura K Günther E. Unimpaired Allorejection of Cells Deficient for the Mannose 6-Phosphate Receptors Mpr300 and Mpr46. Transplantation (2004) 78(5):758–61. doi: 10.1097/01.tp.0000131815.43399.58 15371683

[60] Xia C Braunstein Z Toomey AC Zhong J Rao X. S100 Proteins as an Important Regulator of Macrophage Inflammation. Front Immunol (2017) 8:1908. doi: 10.3389/fimmu.2017.01908 29379499 PMC5770888

[61] Tong L Lan W Lim RR Chaurasia SS. S100a Proteins as Molecular Targets in the Ocular Surface Inflammatory Diseases. Ocul Surf (2014) 12(1):23–31. doi: 10.1016/j.jtos.2013.10.001 24439044

[62] Wilkinson A Kawaguchi N Geczy C Di Girolamo N. S100a8 and S100a9 Proteins Are Expressed by Human Corneal Stromal Dendritic Cells. Br J Ophthalmol (2016) 100(9):1304–8. doi: 10.1136/bjophthalmol-2016-308827 27388245

[63] Wang S Song R Wang Z Jing Z Wang S Ma J. S100a8/A9 in Inflammation. Front Immunol (2018) 9:1298. doi: 10.3389/fimmu.2018.01298 29942307 PMC6004386

[64] Park HM Kim YW Kim KJ Kim YJ Yang YH Jin JM. Comparative N-Linked Glycan Analysis of Wild-Type and Alpha1,3-Galactosyltransferase Gene Knock-Out Pig Fibroblasts Using Mass Spectrometry Approaches. Mol Cells (2015) 38(1):65–74. doi: 10.14348/molcells.2015.2240 25518929 PMC4314127

[65] Chihara RK Lutz AJ Paris LL Wang ZY Sidner RA Heyrman AT. Fibronectin From Alpha 1,3-Galactosyltransferase Knockout Pigs Is a Xenoantigen. J Surg Res (2013) 184(2):1123–33. doi: 10.1016/j.jss.2013.04.012 23673165

