# Supplementary material for: Proteomics Analysis of Aqueous Humor and Rejected Graft in Pig-to-Non-Human Primate Corneal Xenotransplantation
Source: Front Immunol. 2022 Mar 24;13:859929. doi: 10.3389/fimmu.2022.859929 (PMC8986976; doi:10.3389/fimmu.2022.859929)
Supplement: Supplementary file 1 [file DataSheet_1.docx]

Supplementary Material

# Supplementary Figure 1


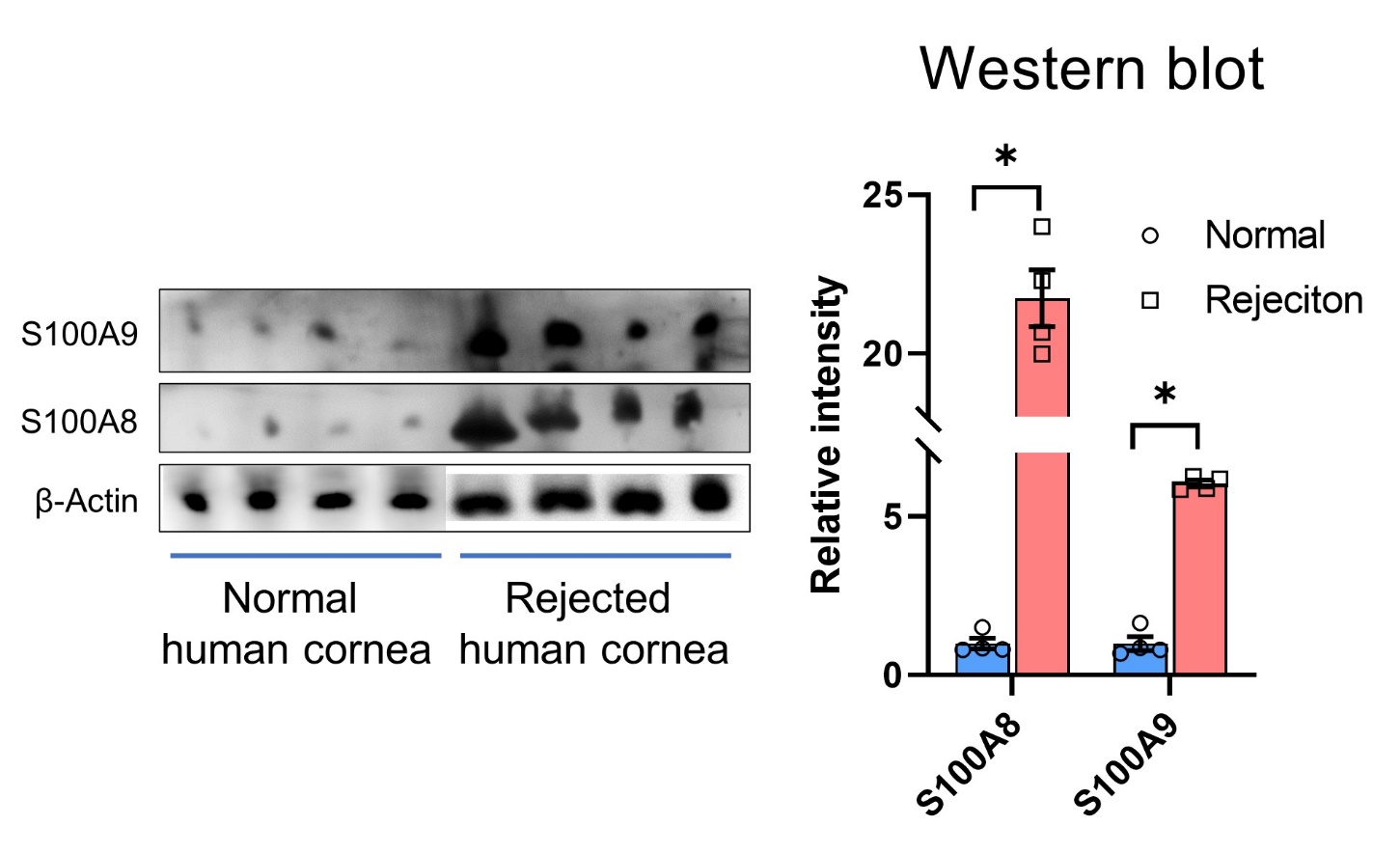


**Supplementary Figure 1.** Western blotting to evaluate the expression of S100A9 and S100A8 in the normal human corneal tissue (n = 4) and rejected human corneal tissue (n = 4). The expression of S100A9 and S100A8 was significantly elevated in rejected corneal tissue, when compared to that in the normal corneal tissue (all p = 0.029; Mann-Whitney test). Data are presented as means ± standard error. Normal corneas for research were provided by the Eversight Eye Bank (Ann Arbor, MI, USA). Seoul National University Hospital institutional review board approved the use of human corneas (No. 1102-092-353)
