# Supplementary material for: Proteomics Analysis of Aqueous Humor and Rejected Graft in Pig-to-Non-Human Primate Corneal Xenotransplantation
Source: Front Immunol. 2022 Mar 24;13:859929. doi: 10.3389/fimmu.2022.859929 (PMC8986976; doi:10.3389/fimmu.2022.859929)
Supplement: Supplementary file 2 [file DataSheet_2.docx]

**Supplementary table**

**Supplementary table 1.** Detailed systemic immunosuppressive regimen for each sample.

| Sample No. | Description | Sample acquisition time (after xenotransplantation, weeks) | Systemic immunosuppressive regimen |
| --- | --- | --- | --- |
| Aqueous humor analysis | | | |
| AH_S1(WT) | AH of survival control 1 (donor: WT) | 19 | Anti‐CD20 Ab^a^, Tacrolimus^c^, IVIG, Basiliximab, Methylprednisolone |
| AH_S2(WT) | AH of survival control 2 (donor: WT) | 21 |  |
| AH_S3(WT) | AH of survival control 3 (donor: WT) | 22 |  |
| AH_S4(WT) | AH of survival control 4 (donor: WT) | 20 | Anti‐CD20 Ab^b^, Tacrolimus^d^, IVIG, Basiliximab, Methylprednisolone |
| AH_S5(WT) | AH of survival control 5 (donor: WT) | 20 |  |
| AH_RO1(WT) | AH of rejection ongoing 1 (donor: WT) | 8 | Anti‐CD20 Ab^b^, Tacrolimus^d^, IVIG, Basiliximab, Methylprednisolone |
| AH_RO2(WT) | AH of rejection ongoing 2 (donor: WT) | 13 |  |
| AH_RO3(WT) | AH of rejection ongoing 3 (donor: WT) | 21 | Tacrolimus^e^, IVIG, Basiliximab, Methylprednisolone |
| AH_RO4(GTKO) | AH of rejection ongoing 4 (donor: GTKO) | 5 | Tacrolimus^e^, IVIG, Basiliximab, Methylprednisolone |
| AH_RO5(GTKO) | AH of rejection ongoing 5 (donor: GTKO) | 10 |  |
| AH_RO6(GTKO) | AH of rejection ongoing 6 (donor: GTKO) | 21 |  |
| AH_R1(WT) | AH of rejection 1 (donor: WT) | 5 | Anti‐CD20 Ab^b^, Tacrolimus^d^, IVIG, Basiliximab, Methylprednisolone |
| AH_R2(WT) | AH of rejection 2 (donor: WT) | 18 |  |
| AH_R3(WT) | AH of rejection 3 (donor: WT) | 24 |  |
| AH_R4(WT) | AH of rejection 4 (donor: WT) | 23 | Tacrolimus^e^, IVIG, Basiliximab, Methylprednisolone |
| AH_R5(GTKO) | AH of rejection 5 (donor: GTKO) | 8 | Tacrolimus^e^, IVIG, Basiliximab, Methylprednisolone |
| AH_R6(GTKO) | AH of rejection 6 (donor: GTKO) | 12 |  |
| AH_R7(GTKO) | AH of rejection 7 (donor: GTKO) | 18 |  |
| Corneal analysis | | | |
| C_S1(WT) | Survived cornea 1 (donor: WT) | 26 | Anti‐CD20 Ab^b^, Tacrolimus^d^, IVIG, Basiliximab, Methylprednisolone |
| C_S2(WT) | Survived cornea 2 (donor: WT) | 67 | Anti‐CD20 Ab^a^, Tacrolimus^d^, IVIG, Basiliximab, Methylprednisolone |
| C_R1(WT) | Rejected cornea 1 (donor: WT) | 22 | Tacrolimus^e^, IVIG, Basiliximab, Methylprednisolone |
| C_R2(WT) | Rejected cornea 2 (donor: WT) | 25 |  |
| C_R3(GTKO) | Rejected cornea 3 (donor: GTKO) | 19 | Tacrolimus^e^, IVIG, Basiliximab, Methylprednisolone |
| C_R4(GTKO) | Rejected cornea 4 (donor: GTKO) | 23 |  |

Note. All groups for topical immunosuppressants: All NHPs received topical prednisolone acetate 1% (Pred forte^®^; Allergan, Irvine, CA, USA) daily for 3 months and injected subconjunctivally with dexamethasone 1.5 mg/0.3 mL (JW Pharmaceutical, Seoul, Republic of Korea) every week. Anti‐CD20 Ab (Rituximab; MabThera^®^, Hoffmann‐La Roche, Basel, Switzerland) was intravenously administered at a dose of 20 mg/kg on postoperative days 0 and 7, and every 2^a^ or 3^b^ months. (Am J Transplant. 2018;18:2330‐2341.; Xenotransplantation. 2018;25:e12442)
Tacrolimus (Prograf^®^; Astellas Pharma US, Deerfield, IL, USA) was intramuscularly administered twice daily at a dose of 0.05^c^ or 0.035^e^ mg/kg or at a dose of 0.05 mg/kg for 4 weeks followed by 0.035 mg/kg^d^.
IVIG, basiliximab, and methylprednisolone were used with the same protocol in all groups. IVIG was intravenously administered on preoperative day 1 and postoperative day 14 at a dose of 1 g/kg. Basiliximab was intravenously administered at a dose of 0.3 mg/kg on postoperative days 0 and 4. Methylprednisolone was intramuscularly administered at an initial dose of 2 mg/kg/d and tapered over 5 weeks.
AH, aqueous humor; S, survival; RO, rejection ongoing; R, rejected; WT, wild type; GTKO, α-1,3-galactosyltransferase gene-knockout
